# Quantitative identification of proteins that influence miRNA biogenesis by RNA pull-down–SILAC mass spectrometry (RP–SMS)

**DOI:** 10.1016/j.ymeth.2018.06.006

**Published:** 2019-01-01

**Authors:** Nila Roy Choudhury, Gracjan Michlewski

**Affiliations:** a Wellcome Trust Centre for Cell Biology, University of Edinburgh, Michael Swann Building, Edinburgh EH9 3BF, UK; b Division of Infection and Pathway Medicine, University of Edinburgh, The Chancellor’s Building, 49 Little France Crescent, Edinburgh EH16 4SB, UK; c Zhejiang University-University of Edinburgh Institute, Zhejiang University, 718 East Haizhou Road, Haining, Zhejiang 314400, PR China

**Keywords:** RNA, RNA-binding protein, miRNA, miRNA biogenesis, RNA pull-down, Mass spectrometry

## Abstract

RNA-binding proteins mediate and control gene expression. As some examples, they regulate pre-mRNA synthesis and processing; mRNA localisation, translation and decay; and microRNA (miRNA) biogenesis and function. Here, we present a detailed protocol for RNA pull-down coupled to stable isotope labelling by amino acids in cell culture (SILAC) mass spectrometry (RP–SMS) that enables quantitative, fast and specific detection of RNA-binding proteins that regulate miRNA biogenesis. In general, this method allows for the identification of RNA-protein complexes formed using *in vitro* or chemically synthesized RNAs and protein extracts derived from cultured cells.

## Highlights

• RNA pull-down SILAC mass spectrometry (RP–SMS) identifies miRNA biogenesis factors.
• Pre-let-7a-1 binds a number of RNA-binding proteins.
• GGAG and UAGG motifs are confirmed to bind LIN28A and hnRNP A1, respectively.

## 1 Introduction

miRNAs regulate gene expression and control a variety of biological processes, including developmental timing, differentiation, metabolism and neuronal patterning [1], [2], [3], [4]. Importantly, a number of miRNAs are expressed in a tissue-specific manner, thereby contributing to cell differentiation and function [5]. Moreover, changes in the levels of a few miRNAs affect processes that include neural differentiation and the formation of induced pluripotent stem cells [6], [7]. Finally, aberrant miRNA expression is linked to a variety of human pathological states including the initiation, progression and metastasis of cancer [8], [9].

Mature miRNAs are derived from primary miRNAs (pri-miRNAs) by nuclear and cytoplasmic processing events [10]. In the nucleus, Drosha [11] and its RNA-binding partner, DGCR8 [12], [13], [14], [15], convert pri-miRNAs to stem-loop precursor miRNAs (pre-miRNAs) that are exported to the cytoplasm by Exportin 5 [16]. In the cytoplasm, Dicer cleaves off the pre-miRNA terminal loop, leaving a miRNA duplex [4], [17]. Subsequently, one strand of this duplex is incorporated into the RNA-induced silencing complex (RISC), which binds its target mRNA(s) by imperfect base-pairing, followed by translational repression and, possibly, deadenylation and degradation of the targeted mRNAs [18], [19].

We have previously shown that hnRNP A1, a protein implicated in many aspects of RNA processing, binds to the conserved terminal loop of pri-miRNA-18a [20], [21] and pri-let-7a-1 [22], stimulating and inhibiting their processing, respectively. Since then, the biogenesis of many miRNAs has been shown to be regulated by various RNA-binding proteins (RBPs) [23], [24]. We have elucidated the mechanisms that regulate miRNA biogenesis in mammalian cells using a method that combines RNA pull-down with stable isotope labelling by amino acids in cell culture (SILAC) high-throughput mass spectrometry (which we call here RP–SMS), developed in our laboratory. We have identified RBPs that regulate the production of brain-enriched and brain-specific miRNAs, such as miRNA-7 and miRNA-9 [25], [26], [27], as well as factors responsible for the selective uridylation and degradation of miRNA precursors in embryonic cells [28]. Here, we describe a step-by-step RP–SMS protocol that can be used to identify miRNA biogenesis factors as well as any other RNA-protein complexes.

## 2 Materials and equipment

### 2.1 SILAC

- SILAC media, heavy and light (e.g. R6K4 ‘heavy’ and R0K0 ‘light’ (DC Biosciences))
- Dialysed calf serum (DC Biosciences)
- SDS-polyacrylamide gel (we use NuPAGE™ 4–12% Bis-Tris Protein Gels, Invitrogen, Thermo Fisher Scientific)
- NuPAGE LDS Sample Buffer (Invitrogen)
- NuPAGE Sample Reducing Agent (Invitrogen)
- NuPAGE MOPS SDS Running Buffer (Invitrogen)
- NuPAGE Antioxidant (Invitrogen)
- GelCode Blue Safe protein stain (Thermo Fisher Scientific)
- Razor blade
- 50 mM Ammonium bicarbonate (ABC) (Sigma)
- Acetonitrile (Thermo Fisher Scientific)
- 10 mM DTT (Sigma)
- 55 mM Iodoacetamide (IAA) (Sigma)
- 20 μg Trypsin glass vial (Thermo Fisher Scientific)
- 0.1% Trifluoroacetic acid (TFA) (Sigma)
- 10% TFA (Sigma)
- Trypsin buffer (Add 20 μl 0.1% TFA to 20 μg trypsin. Mix 100 μl acetonitrile (final concentration 10%), 200 μl 50 mM ABC (final concentration 10 mM) and 700 μl water. Add 3 μl of trypsin solution to 227 μl acetonitrile/ABC solution to make Trypsin buffer).
- Stage tips C18 (Sigma Supelco)
- Methanol (Fisher Scientific)
- Mass spectrometer

### 2.2 *In vitro* transcription

- Primers for your RNA of interest, including a forward primer that contains the T7 promoter sequence TAATACGACTCACTATAGG followed by 20 nt of sense DNA template
- High-fidelity PCR kit (such as Phusion polymerase, Thermo Fisher Scientific)
- PCR machine
- T7 RNA polymerase (such as NxGen T7 RNA Polymerase, Lucigen)
- rNTPs 20 mM (Roche)
- RNaseOUT (Invitrogen)
- TURBO DNase (Ambion)
- 3 M Sodium acetate (NaOAc) (Fisher Scientific)
- 100% Ethanol (EtOH) (Fisher Scientific)
- 70% EtOH
- Urea loading buffer (ULB) (20 mM EDTA, 7 M Urea, bromophenol blue, xylene cyanol)
- 10% polyacrylamide gel (Acrylamide Bis-Acrylamide 19:1, Severn Biotech) containing 7 M Urea (Fisher Scientific)
- 1X Tris/Borate/EDTA (TBE) buffer (To make 5X TBE buffer, dissolve 54 g Tris base and 27.5 g boric acid in approximately 900 ml of deionized water. Add 20 ml 0.5 M EDTA (pH 8.0), and bring the final volume to 1 L using deionized water)
- Stains All (Sigma-Aldrich)
- RNA extraction solution (0.3 M NaOAc pH 5.2 (Fisher Scientific), 0.5 mM EDTA (Fisher Scientific), 0.1% w/v SDS (Fisher Scientific))

### 2.3 RNA pull-down

- *In vitro*-transcribed and purified RNA (500 pmol)
- Adipic acid dihydrazide-Agarose (Sigma)
- 3 M NaOAc pH 5 (Fisher Scientific)
- 0.1 M NaOAc pH 5
- 0.1 M Sodium (meta) periodate (Sigma)
- 100% EtOH
- 70% EtOH
- 4 M KCl (Fisher Scientific)
- 2 M KCl
- Roeder D buffer (100 mM KCl, 20% (v/v) glycerol (Fisher Scientific), 0.2 mM EDTA (Fisher Scientific), 100 mM Tris pH 8.0 (Invitrogen), 0.5 mM DTT (Thermo Fisher Scientific), 0.2 mM PMSF (Sigma))
- 100 mM MgCl_2_ (VWR)
- 0.5 M Sodium creatine-phosphate dibasic tetrahydrate (CIP) (Sigma)
- 100 mM ATP (Sigma)
- RNaseOUT (Invitrogen)
- Buffer G (20 mM Tris-HCl pH 7.5, 137 mM NaCl (Fisher Scientific), 1 mM EDTA, 1% TritonX-100 (Fisher Scientific), 10% glycerol, 1.5 mM MgCl_2_ (Fisher Scientific), 1 mM DTT, 0.2 mM PMSF (Thermo Fisher Scientific))
- NuPAGE LDS Sample Buffer (Invitrogen)
- NuPAGE Sample Reducing Agent (Invitrogen)
- SDS-polyacrylamide gel (we use NuPAGE™ 4–12% Bis-Tris Protein Gels, Invitrogen)
- NuPAGE MOPS SDS Running Buffer (Invitrogen)
- NuPAGE Antioxidant (Invitrogen)
- GelCode Blue Safe protein stain (Thermo Fisher Scientific)

## 3 Protocol

### 3.1 Overview

By combining RNA pull-down with SILAC [29] high-throughput mass spectrometry (RP–SMS) we can identify proteins that bind specifically to a given RNA (such as a pri-miRNA or pre-miRNA), as opposed to non-specifically to beads [25]. SILAC is a method that detects protein abundance using the incorporation of non-radioactive ‘heavy’ amino acid isotopes, which can be distinguished from naturally occurring ‘light’ amino acid isotopes by mass spectrometry. Importantly, we can also detect RNA-protein complexes derived from different cell types or the same cell type exposed to different environmental conditions [26], [27]. Finally, using RP–SMS, we can compare two RNAs (e.g. with or without one or more mutations, or with or without chemical modification) and uncover differentially bound proteins [28].

The method involves producing cell extracts derived from SILAC-labelled cells, obtaining RNA (by *in vitro* transcription or chemical synthesis), performing the RNA pull-down assay, and undertaking mass spectrometry analysis.

### 3.2 SILAC labelling

#### 3.2.1 Incorporate cells with heavy or light isotopes

Grow cells in SILAC media, ‘heavy’ and ‘light’, supplemented with dialysed calf serum, for six passages. We use DMEM medium containing ^13^C-labelled arginine and ^2^D-labelled lysine (R6K4 (DC Biosciences)) as the heavy SILAC medium, and control DMEM containing unlabelled arginine and lysine (R0K0 (DC Biosciences)) as the light SILAC medium.

#### 3.2.2 Verify incorporation of heavy isotopes

##### 3.2.2.1 In-gel digestion

- Electrophorese the protein extract (diluted in 1X NuPAGE LDS Sample Buffer and 1X NuPAGE Sample Reducing Agent) into an SDS-polyacrylamide gel using 1X NuPAGE MOPS SDS Running Buffer supplemented with 1:1000 NuPAGE Antioxidant. Stain the gel with GelCode Blue.
- Using a razor blade, remove an approximately 0.5-cm piece of gel from the centre of the protein lane and cut into small pieces that are approximately 1 mm × 1 mm.
- Transfer the gel pieces to a microfuge tube with 200 μl 50 mM ABC and incubate at room temperature (RT) for 5 min. Remove liquid, add 200 μl acetonitrile, and incubate at RT for 5 min. Repeat the ABC and acetonitrile washes until the solution is no longer blue.
- Add enough 10 mM DTT to cover the pieces, using 20–50 μl or more, and leave at 37 °C for 30 min.
- Remove the liquid, and add 200 μl acetonitrile for 5 min.
- Turn off the light. Remove the acetonitrile, add 55 mM IAA to cover the gel pieces, and incubate at RT for 20 min in the dark. Remove the IAA, add 50 mM ABC for 5 min, and then acetonitrile for 5 min.
- On ice, prepare Trypsin buffer.
- Add Trypsin buffer to just cover the gel pieces, and incubate on ice for 15 min. As the gel absorbs the buffer, add more Trypsin buffer to slightly above the gel pieces, and incubate at 37 °C for 30 min. Add enough acetonitrile/ABC solution to slightly above gel pieces (to make sure the gel pieces do not dry out), and leave at 37 °C overnight.
- Add 1:1 vol of 0.1% TFA to samples (e.g. 100 μl), and leave 20 min. Check that the pH is 1–3, or adjust by adding 10% TFA (generally, 0.5 μl of 10% TFA is required).
- Activate a stage tip C18 by pushing or spinning 20 μl methanol through the tip. Next, condition the tip by pushing or spinning 40 μl 0.1% TFA through the tip.
- Load the sample onto the tip by spinning. Wash by pushing or spinning 60 μl 0.1% TFA through the tip.
- Maintain the stage tip at −20 °C until ready to load onto a mass spectrometer.

##### 3.2.2.2 Mass spectrometry

- Following digestion and peptide purification, perform an LC-MS/MS analysis using an orbitrap mass spectrometer or a similar machine. Determine the efficiency of heavy label incorporation into peptides by manually examining randomly selected peptides from the raw file. Since arginine and lysine may have different labelling efficiencies, this needs to be done separately for arginine- and lysine-containing peptides. Arginine-to-proline conversion should also be examined.

### 3.3 RNA synthesis

#### 3.3.1 PCR

- Design primers that encompass your RNA of interest, appending a T7 promoter sequence (TAATACGACTCACTATAGG) to the 5′ end of the forward primer. Amplify the DNA using high-fidelity PCR, and confirm the size of the PCR product by electrophoresis in an agarose gel.

#### 3.3.2 *In vitro* transcription

- Mix the following in a tube. We use NxGenT7 RNA Polymerase.

**Table:** 

DNA	100 μl
10× buffer	25 μl
rNTPs 20 mM	12.5 μl
RNaseOUT	5 μl
H_2_0	97.5 μl
T7 RNA polymerase	10 μl
	Total 250 μl

- Incubate at 37 °C for 1.5 h.
- Add 1–2 μl TURBO DNase, and incubate at 37 °C for 10 min.
- Add 1/10 vol of 3 M NaOAc and 3 × volume of 100% EtOH, and precipitate on dry ice for 30 min or at −20 °C overnight.
- Precipitate by spinning at 4 °C and full speed for 20 min. Wash the pellet with 70% EtOH, spinning 3 min at full speed, and re-suspend in 40 μl water and 40 μl ULB.
- Boil at 90 °C for 2 min, and subsequently cool for 2 min on ice.
- Split the sample into two, and electrophorese each half in separate wells of a pre-run, warm 10% polyacrylamide/urea gel using 1X TBE as a running buffer.
- Stain with Stains All, and cut out the bands.
- Add 300 μl RNA extraction solution, and leave on the bench overnight.
- Next day, transfer the supernatant to a new tube, and add 900 μl 100% EtOH. Incubate at −20 °C over-night, or on dry ice for 1 h.
- Spin at full speed for 20 min to precipitate the RNA, and wash the resulting RNA pellet with 70% EtOH.

### 3.4 RNA pull-down

#### 3.4.1 Coupling of RNA to agarose beads

- Prepare the following mix in a total volume of 200 μl:

**Table:** 

RNA	500 pmol
NaOAc	6.7 μl 3 M NaOAc pH 5 (final concentration 100 mM)
Sodium (meta) periodate	10 μl 0.1 M (final concentration 5 mM)
H_2_0	to 200 μl

- Wrap all tubes in foil to protect from light. Leave rotating on a wheel at RT for 1 h.
- Precipitate the RNA by adding 15 μl 3 M NaOAc and 600 μl 100% EtOH, and leave on dry ice for 10–30 min. Spin at 4 °C and full speed for 20 min. Wash with 1 ml 70% EtOH, and spin at 4 °C and full speed for 3 min. Re-suspend the RNA in 500 μl 100 mM NaOAc pH 5.
- Transfer 250 μl Adipic acid dihydrazide-Agarose resin (50%) per reaction to a 15 ml tube using a cut pipette tip, and wash 3 times with 10 ml 100 mM NaOAc, spinning 300 rpm for 3 min after each wash. Re-suspend the final pellet in enough 100 mM NaOAc to have a mix of 50% beads/liquid. You will need 200 μl mix per reaction. Add 200 μl beads to the 500 μl periodate-treated RNA, and leave rocking at 4 °C overnight.
- Remove the foil. Add 700 μl 4 M KCl to the mix, and rock at RT for 30 min. Pellet the resin at 3000 rpm for 3 min, discarding the supernatant. Wash the resin twice with 1 ml 2 M KCl, and 3 times with Roeder D buffer, spinning at 300 rpm for 2 min after each wash.

#### 3.4.2 Incubate RNA-beads with protein extracts

Add to the pelleted resins:

**Table:** 

Extract	250 μl (1–1.5 mg total protein, add Roeder D buffer if using less protein)
MgCl_2_	9.75 μl 100 mM (1.5 mM)
CIP	32.5 μl 0.5 M (25 mM)
ATP	3.25 μl 100 mM (0.5 mM)
RNaseOUT	5 μl
H_2_0	349.5 μl
	Total 650 μl

- Incubate at 37 °C for 30 min with rocking at 400 rpm. Pellet by spinning at 1000 rpm for 3 min. Save the supernatant as a loading control.

#### 3.4.3 Wash away unbound protein

- Work on ice. Wash reactions three times with 1 ml Buffer G, spinning at 4 °C and 1000 rpm for 2 min. Combine the “light” and “heavy” samples during the last wash.

#### 3.4.4 Electrophorese proteins into a SDS-PAGE gel

- To the beads add: 39 μl H_2_O, 15 μl NuPAGE LDS Sample Buffer, and 6 μl NuPAGE Sample Reducing Agent.
- Boil at 70 °C for 10 min while shaking. Spin at RT and full speed for one min, and load 30 μl.
- Electrophorese the sample into an SDS-polyacrylamide gel using 1X NuPAGE MOPS SDS Running Buffer supplemented with 1:1000 NuPAGE Antioxidant, so that the dye migrates 1 cm into the gel. Stain the gel with GelCode Blue Safe protein stain.

### 3.5 Mass spectrometry analysis

- Cut out the band, proceed with in-gel digestion as described in Section 3.2.2, and perform an LC-MS/MS analysis using an orbitrap mass spectrometer or its equivalent. The MaxQuant software [30] platform is used to analyse the raw mass-spectrometry data in order to determine the ratio of the heavy-labelled peptides to the light-labelled peptides. The samples can also be analysed by Western blotting for known proteins.

## 4 Results

We developed the RP–SMS method to identify proteins that bind to the let-7a-1 pre-miRNA (pre-let-7a-1). Pre-let-7a-1 is known to be regulated by LIN28A [31], [32], [33], hnRNP A1 [20], [22] and KSRP [34]. To determine what protein factors bind specifically to pre-let-7a-1, as opposed to non-specifically to agarose beads, we compared the beads alone incubated with ‘light’ HeLa-cell extract to pre-let-7a-1-coupled beads incubated with ‘heavy’ HeLa-cell extract.

**Fig. 1:**
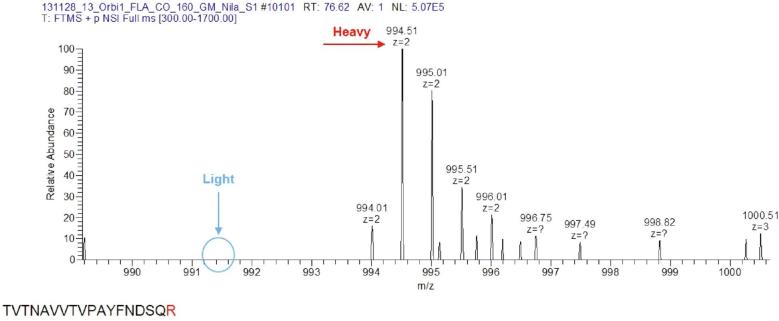
An example of an MS1 spectrum in which the incorporation of heavy arginine is displayed. A peptide with a heavy-labelled arginine is highlighted in red. The absence of the corresponding non-labelled arginine-containing peptide (highlighted in blue) confirms full SILAC incorporation. (For interpretation of the references to colour in this figure legend, the reader is referred to the web version of this article.)

We incorporated R6K4 into HeLa cells constitutively expressing LIN28A, and confirmed the SILAC incorporation (Fig. 1). Protein extracts from ‘heavy’ (H) and ‘light’ (L) cells were generated by scraping adherent cells in Roeder D buffer, followed by cell lysis using sonication, and clearing the extracts using centrifugation. The RP-SMS protocol was followed for the pre-let-7a-1 as described above, providing us with a list of identified proteins and their H/L ratios, peptide counts and intensities, and many additional features (Supplementary Table 1).

Notably, a H/L ratio of 1 in the RNA pull-down compared to the beads-only control signifies that there is no enrichment of protein binding to RNA. H/L ratios of ≤0.5 indicate either preferential cellular protein binding to the beads-only control or protein contaminants that derive from the environment (such as the experimentalist’s skin). H/L ratios of ≥2 indicate specific protein binding to the RNA.

**Fig. 2:**
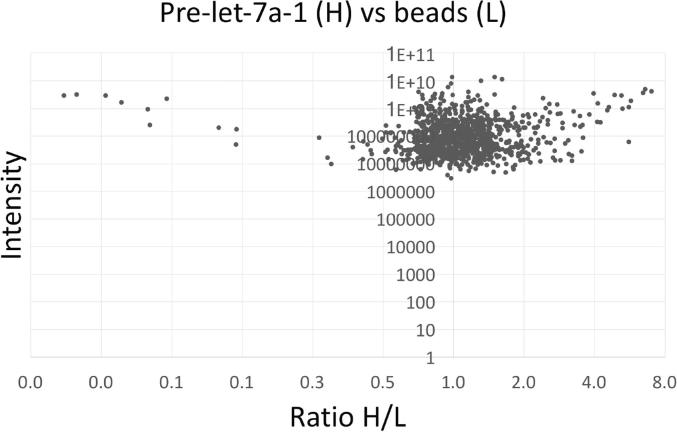
Distribution of H/L ratios among proteins identified in the pre-let-7a-1 pull-down. We used the RP–SMS method to identify protein factors that bind specifically to pre-let-7a-1 (H) or non-specifically to beads alone (L). Results reveal that although most proteins identified do not bind specifically to pre-let-7a-1 (i.e., they have H/L ratio of less than 2), 81 proteins are enriched 2-fold or more in the pre-let-7a-1 pull-down.

H/L ratios revealed that 81 cellular proteins were enriched at least two-fold (H/L ratio ≥2) in the pre-let-7a-1 pull-down compared to the beads-only control (Fig. 2), among which were many known RNA-binding proteins, such as the helicase DHX9, several hnRNP proteins, LIN28A, the splicing factor SRSF1, and the novel RNA-binding protein TRIM25 (Supplementary Table 1). In contrast, the ratio observed for 828 other proteins was >0.5 and <2 when binding to RNA vs. binding to the beads-only control were compared, indicating non-specific binding. Of the 19 proteins enriched on beads alone (H/L ratio ≤0.5), there were several types of keratin (a common mass spectrometry contaminant).

**Fig. 3:**
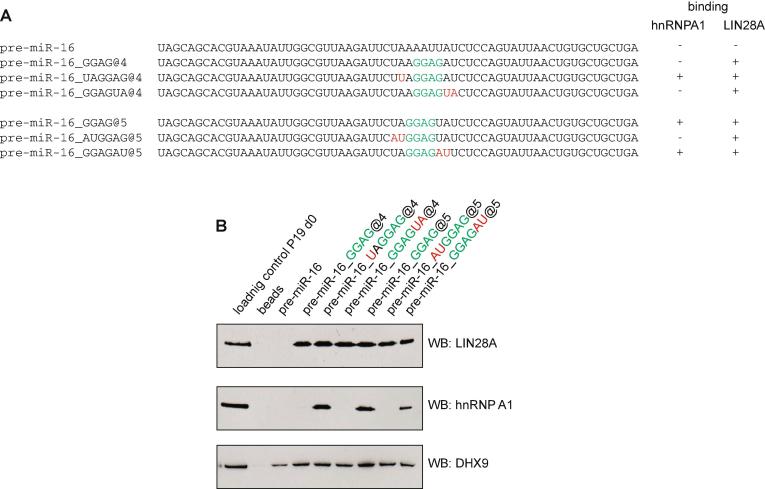
Introduction of LIN28A- or hnRNAP A1-binding sites into pre-miR-16 validates the specificity of the RNA pull-down. (a) A LIN28A-binding site (GGAG) and/or an hnRNP A1-binding site (UAGG) was introduced into pre-miR-16. In set @4, AAUU was changed to GGAG, whereas in set @5, AAAU was changed to GGAG. Pre-miR-16_UAGGAG@4, pre-miR-16_GGAG@5 and pre-miR-16-GGAGAU@5 were engineered to harbor a hnRNP A1 UAGG binding site. Green letters indicate introduced GGAG binding sites. Red letters mark A-to-U or U-to-A substitutions, which either test for the specificity of RNA pull-down, or, in case of pre-miR-16_UAGGAG@4, introduce an hnRNP A1-binding site. b) RNA pull-down and Western blotting were performed. Western blotting probed for LIN28A, hnRNAP A1, or DHX9. Results showed that only the pre-miR-16 mutants that contain a LIN28A-binding site bind LIN28A, and only those mutants that contain an hnRNP A1-binding site bind hnRNP A1. All pre-miR-16 mutants bind DHX9, which binds any double-stranded RNA. (For interpretation of the references to colour in this figure legend, the reader is referred to the web version of this article.)

To validate the specificity of our RNA pull-down assay, we introduced a well-known LIN28A-binding site (GGAG) into the terminal loop of pre-miR-16, which does not bind LIN28A (Fig. 3A) [27], [28], [33], [35]. All pre-miRNA-16 mutants showed efficient LIN28A binding (Fig. 3B). DHX9, which recognises double-stranded RNA, also displayed binding to all pre-miRNA-16 transcripts (Fig. 3B). Finally, we introduced hnRNP A1-binding sites (UAGG) [20], [35] into pri-miR-16 and showed that all mutants with UAGG sequences bind hnRNP A1 efficiently (Fig. 3B). It is important to note that introducing binding sites for miRNA biogenesis factors does not automatically mean that processing of the pri-miRNAs or pre-miRNAs is dependent on or regulated by these factors. Other sequences, structural elements, and protein factors cooperate to control the miRNA processing pathway.

As the next step towards identifying miRNA processing factors, one could take all significant hits from the RP–SMS experiment and perform loss-of-function (RNAi and/or CRISPR/Cas9) or gain-of-function (over-expression) experiments. This should be followed by *in vitro* processing assays and/or *in vivo* miRNA processing analysis using extracts from wild-type, candidate factor-depleted, or candidate factor-overexpressing cells.

## 5 Concluding remarks

The regulation of RNA processing by RBPs is at the functional heart of all cells. RBPs mediate and control gene expression by regulating virtually all steps of RNA metabolism [36], [37]. Consequently, they contribute to cellular homeostasis, development and disease. Furthermore, hundreds of novel RBPs have been recently identified [38], [39], [40], [41]. Many of these proteins have not been previously known for their RNA-binding properties and do not contain canonical RNA-binding domains. This highlights the need for methods that detect physiologically relevant RNA-protein complexes.

Many methods to identify protein binding to specific RNAs have recently been established [42]. We have previously developed a method based on RNA-coupled agarose beads and RNase-assisted elution of RBPs [43]. Furthermore, high-throughput screens for regulators of miRNA processing have revealed numerous sequence-specific factors that bind to miRNA precursors and primary transcripts [24], [35], [44]. The RNA pull-down combined with SILAC high-throughput mass spectrometry (RP–SMS) protocol described in this paper allows for versatile experimental design and high levels of sequence specificity for the quantitative detection of RBPs.
